# The Impact of Coffee and Pasture Agriculture on Predatory and Omnivorous Leaf-Litter Ants

**DOI:** 10.1673/031.013.2901

**Published:** 2013-04-16

**Authors:** Nivia da Silva Dias, Ronald Zanetti, Mônica Silva Santos, Maria Fernanda Gomes Villalba Peñaflor, Sônia Maria Forti Broglio, Jacques Hubert Charles Delabie

**Affiliations:** 1 Embrapa Agroindústria Tropical, Rua Dra. Sara Mesquita 2270, Bairro Pici, 60511-110, Fortaleza, CE, Brazil; 2 Departamento de Entomologia, Universidade Federal de Lavras/UFLA, Caixa Postal 37, 37200-000 Lavras, MG, Brazil; 3 Departamento de Entomologia, Escola Superior de Agricultura “Luiz de Queiroz”-ESALQ/USP, Av. Pádua Dias, 11, Caixa Postal 9, 13418-900 Piracicaba, SP, Brasil; 4 Centro de Ciĉncias Agrárias, Universidad Federal de Alagoas (CECA, UFAL). Campus Delza Gitaí, Rod. BR 104, Km 85, 57.100-000, Rio Largo, AL, Brazil; 5 Laboratório de Mirmecologia, Convênio UESC/CEPLAC, Centro de Pesquisa do Cacau. Rod. Ilhéus/ltabuna, Km 22, 45600-000 ltabuna, BA, Brazil

**Keywords:** agroecosystem, diversity index, fragmentation, Formicidae, habitat impact

## Abstract

Ants are known to function as reliable biological indicators for habitat impact assessment. They play a wide range of ecological roles depending on their feeding and nesting habits. By clustering ants in guilds, it is possible both to assess how agriculture and forest fragmentation can disturb ant communities and to predict the ecological impacts due to losses of a specific guild. This study aimed at determining the impact of non-shaded coffee and pasture agriculture on predatory and omnivorous guilds of leaf-litter ants of Atlantic Forest fragments in Minas Gerais, Brazil. Both coffee and pasture agriculture influenced leaf-litter ant community, although coffee was more disruptive than pasture. Coffee agriculture not only disturbed the diversity of predatory ants, but also negatively affected the number of predatory and omnivorous ants when compared to forest fragments. In contrast, pasture agriculture only disrupted the abundance of predatory ants. Fragment edges skirting crops were negatively affected in terms of leaf-litter ant abundance, but not diversity. Cluster analysis showed that forest fragments were similar irrespective of the cultivation, but the borders were similar to the crop. The study assessed agriculture impact by surveying ant guilds, and revealed that the predatory guild is more susceptible than omnivorous ants.

## Introduction

The study of ant guilds has been shown to provide a predictive understanding of community responses to disturbance (Andersen 1997; King et al. 1998; Leal et al. 2010; Wike et al. 2010). This approach allows habitat assessment based on clustering species according to their ecological roles, instead of focusing on only one or few ant species as biological indicators (Silvestre et al. 2003; Ottonetti et al. 2006). Thus, ant guild surveys are more informative than single or few ant species surveys, but not as extensive and timeconsuming as full ant surveys. Furthermore, habitat impact assessments using guild surveys can simplify the sampling process and decision making on habitat monitoring (Andersen and Majer 2004; Ottonetti et al. 2006). The guild survey model has been successfully employed as a powerful, simple, and economical tool in Australia (Andersen and Majer 2004) and elsewhere (Silvestre and Silva 2001; Ottonetti et al. 2006), mainly for anthropogenic impact assessment (Bestelmeyer and Wiens 1996; Hoffmann et al. 2000; Andersen et al. 2002; Hoffmann and Andersen 2003).

A guild can be defined as a cluster of species that exploits the same types of resources in the habitat and exhibits similar patterns of exploiting resources (Root 1967). Delabie et al. (2000) proposed classification of ant communities in “functional groups” (a notion rather close to the guild concept) based on literature information of leaf-litter ant biology from the Atlantic Forest of south Bahia State, Brazil. Although the two concepts are similar, functional group is more focused on how resources are processed by organisms to provide an ecosystem function, while guild refers more to the mechanisms of resource sharing in a competitive context (Blondel 2003). Silvestre et al. (2003) also used ecological roles as a parameter to describe the ant communities of cerrado vegetation according to the way they exploit and occupy the habitat. Tentative classification of Neotropical ants based on morphological characters and behavioral specialization would complement the guild concept (Brandão et al. 2009; Silva and Brandão 2010).

The disruptive impact of agriculture on leaflitter ant species richness and abundance may be caused by the reduction of canopy cover (Perfecto and Vandermeer 1996; Armbrecht and Perfecto 2003), litter depth, and soil volume (Carvalho and Vasconcelos 1999; Mezger and Pfeiffer 2010). Previous studies have shown that non-shaded plantations are particularly disruptive to ant communities (Nestel and Dickschen 1990; Perfecto et al. 1997; Armbrecht et al. 2005; Philpott et al. 2006), which is also the case in coffee crops and pastures. Traditional coffee plantations usually consist of low biodiversity, and coffee trees are heavily pruned, drastically reducing the canopy cover (Moguel and Toledo 1999). Shade coffee plantations (i.e., raising coffee trees under the shade of larger trees) have been shown to be less disruptive to ant communities because they provide a refuge for the ants (Perfecto et al. 1996). Another serious consequence of agriculture expansion is forest fragmentation, which leads to several abiotic and biotic changes mainly due to area reduction and border extension of fragments. Fragmentation severely impacts the ecosystem by disrupting species richness and composition, population and community dynamic, trophic interactions, and ecological processes (Laurance and Vasconcelos 2009).

Agroforestry, pasture agriculture, and coffee crops have been shown to alter ant species composition and reduce species richness in Brazil (Majer et al. 1997; Vasconcelos 1999; Marinho et al. 2002; Ramos et al. 2004; Dias et al. 2008). Here, our study focused on investigating if predatory and omnivorous ants respond in the same way to coffee and pasture agriculture. We compared the frequency and richness of predatory and omnivorous ants in crop areas, borders, and forest fragments adjacent to coffee crops and pastures. We predicted that agriculture would impact predatory ants, which have more specialized diets, more than omnivorous ants. Thus, predatory ants would be more susceptible to changes in environment, as they are less prone to adapt to alternative food sources. Surveys based on functional groups or guilds will provide a better understanding of how agriculture impacts leaf-litter ant communities, and which ecological functions displayed by ants can be compromised.

## Material and Methods

The study was conducted in coffee plantations and pasture areas skirting semi-deciduous forest fragments inserted into the Atlantic Forest biome (Veloso et al. 1991). The experimental areas were located in the municipalities of Lavras, Ijací, and Perdões, southern Minas Gerais, Brazil (latitude 21° 00′ S to 21° 19′ S, longitude 44° 00′ W to 45° 07′ W).

Two types of cultivations were sampled, namely coffee and pasture crops. The coffee crop was characterized by non-shaded fields, a small sized cultivar (*Coffea arabica* L. cv. Catuai) (Rutacea), and vegetation of homogeneous structure. Although these areas were not herbicide-treated, manual weeding, which occurred regularly from March to May, was efficient in keeping a low number of weeds. The pasture crop, *Brachiaria decumbens* Stapf (Poaceae), contained invasive plants that had been annually controlled by mechanical weeding. For each cultivation, the narrow space comprising the transition between the crop and the forest fragment (treatment called “border”), and the forest fragments, which were remnants of the native semi-deciduous forest and apparently at the same level of conservation (treatment called “fragment”), were also sampled. In this way, six habitats were sampled (crop, border, and fragment for two cultivations), with five replicates per habitat. Each replicate consisted of 15 samples of 1 m^2^ of leaf litter at a minimum distance of 50 m from each other. Sampling always started at 50 m away from the contact between the forest fragment and the agroecosystem, except samples on the border line. In pasture fields, where leaf-litter was scanty and therefore difficult to remove, samples were manually collected using a hoe. Each sample was sifted and placed on a Winkler extractor for 72 hr (Bestelmeyer et al. 2000) in order to separate ants.

The collected individuals were identified by comparing them with the collection from Laboratório de Mirmecologia at Centro de Pesquisas do Cacau/Comissão Executiva do Piano da Lavoura Cacaueira in Ilhéus, Bahia State, Brazil. The classification of the subfamilies was in accordance with Bolton (2003). The identified individuals were stored at Centro de Pesquisas do Cacau/Comissão Executiva do Piano da Lavoura Cacaueira and the Laboratório de Entomologia Florestal da Universidade Federal de Lavras, Minas Gerais, Brazil). Species were grouped in guilds (omnivorous or predatory) in accordance with Delabie et al. (2000), Silvestre et al. (2003), and Brandão et al. (2009). For each replicate, the total number of omnivorous and predatory ants found in the 15 samples were counted, and the mean number of ants in each habitat was calculated based on the five replicates. Kolmorogov-Smirnov tests were performed in order to rule out heteroscedasticity of error variance and confirm normality of the data. The frequency data of omnivorous and predatory ants answered the parametric requirements, and then were analyzed by twoway analysis of variance and Tukey's HSD test (*p* ≤ 0.05) in order to examine the effect of each independent variable (cultivation and habitat as fixed factors) and their interaction. Also, cluster analysis based on Euclidean distance was performed for the numbers and frequency of omnivorous and predatory species found in each habitat. To assess diversity, Shannon-Wiener (*H*) index was estimated and analyzed in the same way as the frequency data. For all analysis, the software BioStat version 2009 (http://www.analystsoft.com) for Windows® was used.

## Results

A total of 70 omnivorous ant species and 55 predatory ant species were found, comprising 31 genera and 10 Neotropical subfamilies (Table 1). The main subfamilies of predatory ants were Ectatomminae and Ponerinae, whereas Formicinae and Myrmicinae represented the majority of the omnivorous guild in the study area.

Some ant species were exclusively found in a given habitat (Table 2), except in edge areas (border) between the forest fragment and coffee plantation. Although more omnivorous ant species were collected than predatory (Table 1), there was a higher number of exclusive predatory ant species (Table 2). Pasture plantations had the highest number of exclusive predatory and omnivorous (especially *Pheidole* spp.) ant species, even when compared to forest fragments near pasture (Table 2). Some of the exclusive predatory ants found in pastures are army ants, such as *Neivamyrmex* sp., or have an army-like behavior (e.g., *Simopelta* sp.) (Table 1). These ants are generally nomadic (Delabie et al. 2000), so these results should be carefully interpreted since nomadic species may be seasonally found in those habitats. Yet, *Gnamptogenys* and *Anochetus,* also found exclusively in the pastures, comprise litter generalist predators. These ants can also collect nectar from the vegetation and therefore are more likely to survive in disturbed habitats by feeding on alternative food sources (Delabie et al. 2000). The high number of exclusive ants species found in the pasture is in accordance with Vasconcelos (1999) and Kotze and Samways (1999), who found particular and richer leaf-litter ant diversity in pastures when compared to forests. However, only forest fragments had exclusively the specialist predatory ants *Amblyopone* sp.1 and *Discothyrea sexarticulata* Borgmeier (Table 2).

**Figure 1.**
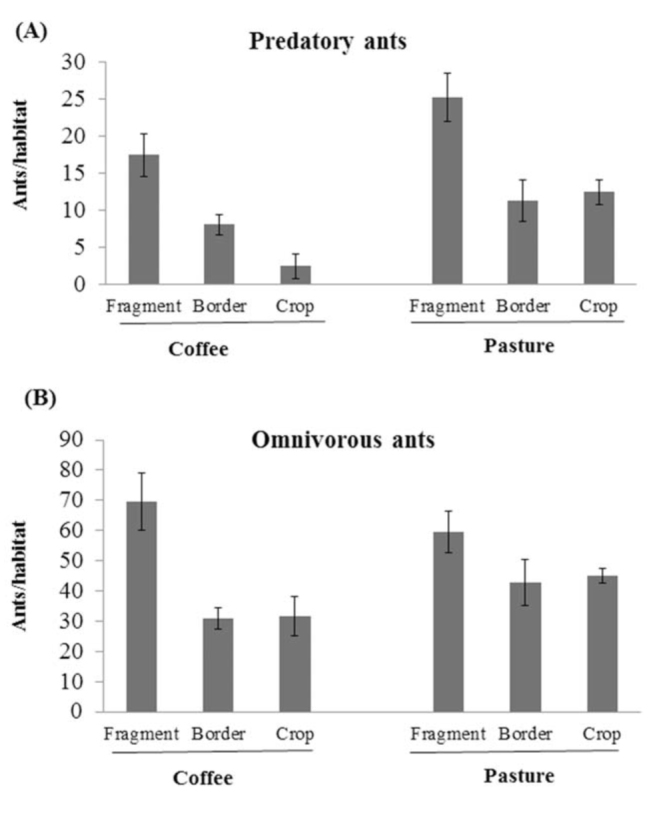
Mean number of ants collected (± SE) in the forest fragments (fragment- Coffee and Pasture), border fragment-coffee and fragment-pasture (Border- Coffee and Pasture) and coffee and pasture agroecosystems (Crop- Coffee and Pasture), (a) Predatory guild; (b) Omnivorous guild. High quality figures are available online.

The diversity of predatory ants, according to Shannon-Wiener diversity index, was affected by cultivation, habitat, and their interaction, and was particularly lower in the coffee habitat (Table 3 and 4). The pairwise comparisons showed that predatory ant diversity was significantly lower in crops compared to borders and forest fragments (Table 5). Within coffee plantations, predatory-ant diversity index was lower in the crop compared to the border and the fragment, although within the pasture, none of the pairwise comparisons were significant. The interaction of habitat and cultivation was significant only for the predatory-ant diversity index in crops (Table 5). On the other hand, the omnivorous ant diversity index was not altered in either coffee or pasture in function of the cultivation and habitat (Table 4). The number of predatory ants differed based on cultivation and habitat, but their interaction term was not significant (Figure 1A, Table 6). In regard to the omnivorous guild, only habitat influenced the number of ants (Figure 1B, Table 6). The pairwise comparison showed there were more predatory ants in the fragments than in the crop and the border in both coffee and pasture cultivations, while omnivorous ants were more numerous in the fragment than in the crop and the border only in coffee cultivation (Figure 1, Table 7).

**Figure 2.**
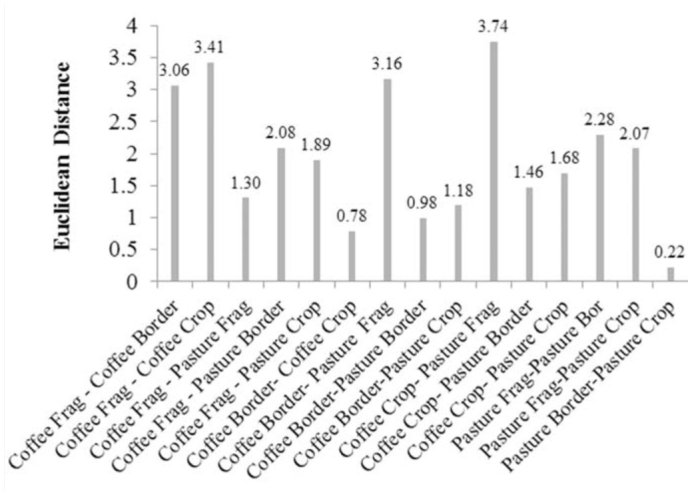
Euclidean distance between all combinations of habitats (frag = fragment, border, and crop) from coffee and pasture cultivations considering number and frequency of predatory and omnivorous ant species. High quality figures are available online.

**Figure 3.**
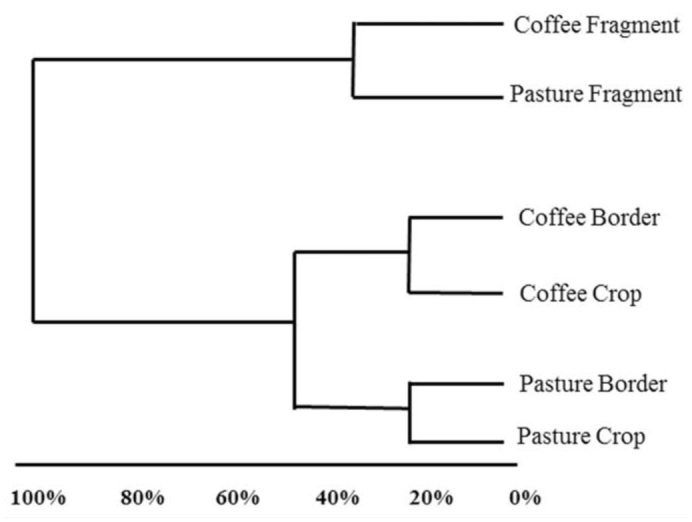
Cluster analysis of habitats (fragment, border, and crop) from coffee and pasture cultivations using Euclidean distance for number and frequency of predatory and omnivorous ant species. High quality figures are available online.

According to the cluster analysis (Figure 2, 3), three clusters were distinguished considering the number and frequency of predatory and omnivorous ant species. Forest fragments of coffee and pasture areas formed one group separated from the other habitats. The other two clusters comprised the border and crop of coffee and pasture plantations, indicating the influence of crops on the border skirting forest fragments (Figure 3). The coffee crop and the forest fragments close to pasture areas were the most distant habitats in terms of ant guild composition, while the pasture and its border with the forest were the most similar (Figure 2). Furthermore, the fact that the leaf-litter ant community was similar in the forest fragments irrespective of the crop points out the importance of the cultivation as a factor modulating predatory and omnivorous ant guild composition.

## Discussion

According to the results, both coffee and pasture agriculture influenced leaf-litter ant community, although coffee was more disruptive than pasture. Coffee agriculture not only disturbed the diversity of predatory ants, but also negatively affected the number of predatory and omnivorous ants when compared to forest fragments. Similarly, Philpott et al. (2006) found that predatory ant diversity was reduced in coffee plantations; however, in their study, the abundance was unaffected. Yet, pasture apparently did not cause any negative effect on leaf-litter ant diversity, but it disrupted the abundance of predatory ants, which was lower in the crop than in the fragment.

Management in coffee and pasture agroecosystems is different. Many insecticides sprayed on coffee plantations are not selective and thus affect all insects (Silva et al. 2005). Considering that pests affect quality of coffee grains, producers often apply insecticides to avoid product depreciation and losses caused by insect damage. Plantations of *B. decumbens* are destined to grazing animals and cannot be constantly treated by insecticides. Also, the pasture areas had not been treated with herbicides, allowing the growth of other plants that provide nectar, an alternative food source for both predatory and omnivorous ants, and the maintenance of the litter on the soil.

The possible mechanisms related to losses in ant diversity and abundance due to coffee intensification are related to physiological factors, such as microclimatic changes, mainly temperature and moisture, or ecological factors, such as availability for nesting sites and food, or invasion by exotic ants, competitive exclusion by aggressive ants, and action of natural enemies (Philpott and Armbrecht 2006).

The edge effect is usually negative to leaflitter ant richness in fragmented habitats (Majer et al. 1997; Sobrinho and Schoereder 2006). Although, in our study, ant species richness in borders was similar to forest fragments, the numbers of ants in the border were, in general, lower than in the fragments and similar to the crops. In the pasture, there was no border effect on omnivorous ants, and even some exclusive omnivorous species were found in the border, such as *Camponotus melanoticus,* which is predominately arborous, and *Pheidole* sp. 24. The results also show that the predatory ant guild is more sensitive to agriculture impact than the omnivorous guild. The lesser sensitivity of omnivorous ants is most likely because they have a wider diet breadth than predatory ants, allowing them to survive by feeding on alternative sources. Hence, as mentioned above, the exclusive predatory ants in pasture or coffee are generalist predators that complement their diet by licking nectar from plants, and therefore might be more resistant to disturbed habitats.

The ultimate consequence of the reduced diversity and frequency of leaf-litter ants in coffee crops would be the disruption of ecological associations (Armbrecht et al. 2005). Predatory ants can play an important role in biological control of economically important pests (Way and Khoo 1992). Even though the results of the present study show that the predatory ant guild composition and frequency have been disrupted by agriculture activity, it cannot be predicted if predation of pests in the agroecosystems is also disrupted for two main reasons. First, an increasing number of predatory ants is not necessarily correlated with an enhanced functional role of ants as predators (Huston 1997). Second, predatory ants can function either as predators or as cryptic herbivores by hemipteran-tending (Philpott and Armbrecht 2006). Tending ants feed on the honeydew excreted by hemipterans and protect hemipterans from natural enemies (Way 1963). Theoretically, this association would be unwanted for agricultural management, as ants benefit hemipteran pests. However, it is still controversial if the mutualistic ant-hemipteran association harms or benefits plants, because it will depend on how predatory ants can suppress other non-honeydew-producing herbivores and the damage caused by hemipterans (Styrsky and EuEubanks 2007).

This study revealed that non-shaded coffee plantations in Brazil cause strong disturbance to both predatory and omnivorous leaf-litter ants, while pasture agriculture only affects predatory ants. A loss in biodiversity can incur in changes in the whole ecosystem, and implementing more sustainable crops, such as shaded-coffee, would help to reduce the impact. To conclude, the findings of this study suggest that the predatory ant guild might be considered as a biological indicator for agricultural impact assessment.

**Table 1.**
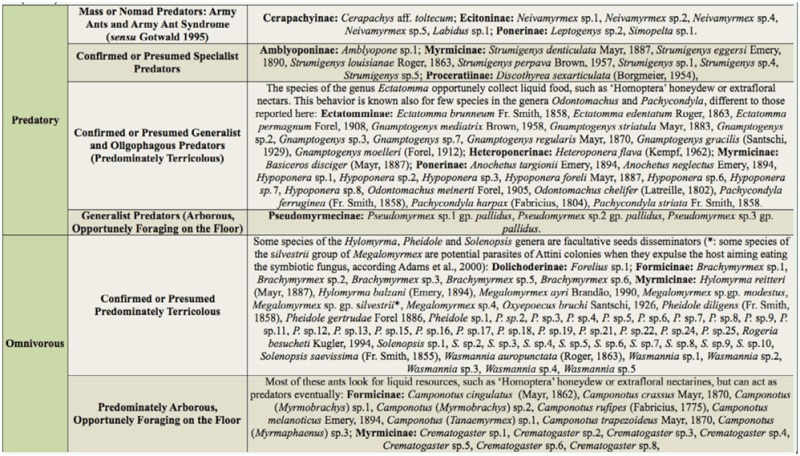
Classification of predatory and omnivorous leaf-litter ant species collected in forest fragments, fragment-coffee and fragment-pasture borders, and coffee and pasture crops, according to Delabie et al. (2000), Silvestre et al. (2003), and Brandão et al. (2009).

**Table 2.**
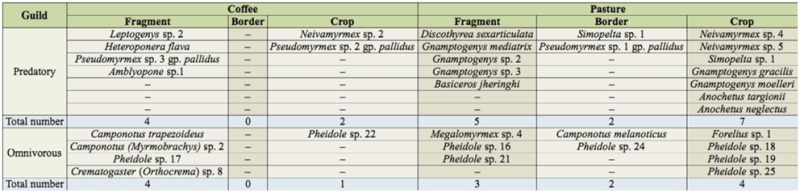
Diversity index of Shannon-Wiener (H') for predatory and omnivorous leaf-litter ants collected in forest fragments (fragment- Coffee and Pasture), border fragment-coffee and fragment-pasture (Border- Coffee and Pasture), and coffee and pasture agroecosystems (Crop- Coffee and Pasture)

**Table 3.**
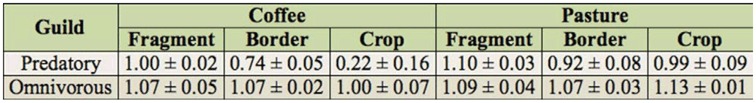
Diversity index of Shannon-Wiener (H') for predatory and omnivorous leaf-litter ants collected in forest fragments (fragment- Coffee and Pasture), border fragment-coffee and fragment-pasture (Border- Coffee and Pasture), and coffee and pasture agroecosystems (Crop- Coffee and Pasture)

**Table 4.**
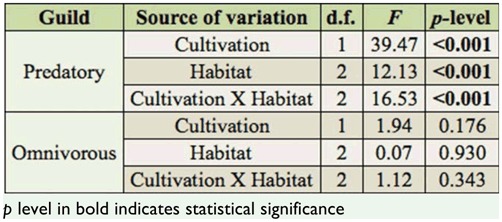
Statistical data of two-way ANOVA considering cultivation (coffee and pasture) and habitat (border, fragment, crop) as variables to analyze the diversity index of Shannon-Wiener (H') of predatory and omnivorous guilds.

**Table 5.**
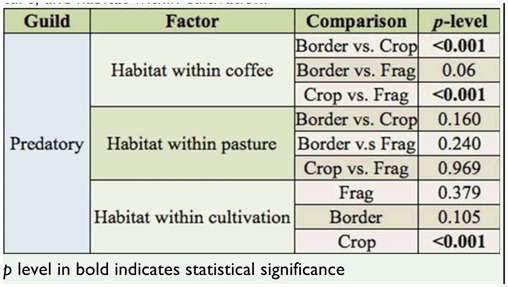
Pairwise comparisons using Tukey's HSD test to analyze the data of the diversity index of Shannon-Wiener (H') for the predatory guild in function of habitat within coffee and pasture, and habitat within cultivation.

**Table 6.**
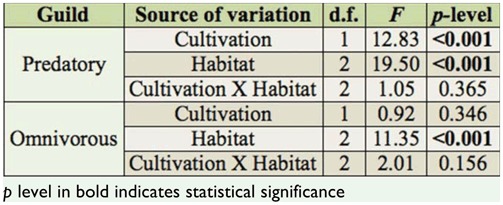
Statistical data of two-way ANOVA considering cultivation (coffee and pasture) and habitat (border, fragment, crop) as variables to analyze the number of predatory and omnivorous ants.

**Table 7.**
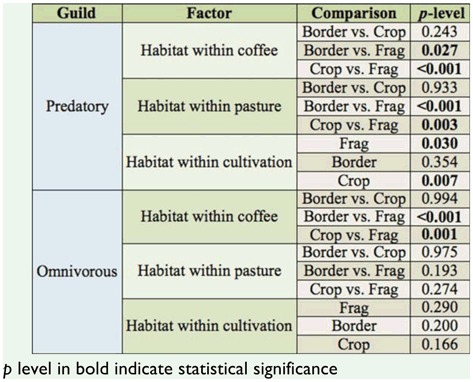
Pairwise comparisons using Tukey's HSD test to analyze the data of the number of predatory and omnivorous ants within the habitat within coffee, the habitat within pasture, and the habitat within cultivation.
